# Soft-tissue vibration and damping response to footwear changes across a wide range of anthropometrics in running

**DOI:** 10.1371/journal.pone.0256296

**Published:** 2021-08-17

**Authors:** Anja-Verena Behling, Marlene Giandolini, Vinzenz von Tscharner, Benno Maurus Nigg

**Affiliations:** 1 School of Human Movement and Nutrition Sciences, The University of Queensland, Brisbane, Queensland, Australia; 2 Biomedical Engineering, Schulich School of Engineering, University of Calgary, Calgary, Alberta, Canada; 3 Human Performance Laboratory, Faculty of Kinesiology, University of Calgary, Calgary, Alberta, Canada; 4 Amer Sports Footwear Innovation and Sport Science Lab, Salomon SAS, Annecy, France; National Tsing Hua University, TAIWAN

## Abstract

Different factors were shown to alter the vibration characteristics of soft-tissue compartments during running. Changing pre-heel strike muscle activation or changing footwear conditions represents two possibilities to influence the vibration response via frequency shift or altered damping. Associated with the study of muscle pre-tuning is the difficulty in quantifying clean experimental data for the acceleration of soft-tissue compartments and muscle activities in heterogeneous populations. The purpose of this study was to determine the vibration and pre-tuning response to footwear across a wide range of participants during running and establish and describe groups formed according to the damping coefficient. 32 subjects were used for further analysis. The subjects ran at a self-selected speed (5 min) on a treadmill in two different shoes (soft & hard), while soft-tissue accelerations and muscle activation at the gastrocnemius medialis were quantified. Damping coefficients, total muscle intensity and dominant vibration frequencies were determined. Anthropometrics and skinfold measurements of the lower limbs were obtained. According to the damping coefficient response to the footwear intervention, three groups were formed, with most runners (n = 20) showing less damping in the hard shoe. Total muscle intensity, anthropometrics, and dominant vibration frequency across footwear were not different for these three groups. Most runners (84.4%) used the strategy of adjusting the damping coefficients significantly when switching footwear. Despite damping being the preferred adjustment to changes in footwear, muscle pre-tuning might not be the only mechanism to influence damping as previously suggested. Future studies should focus on the subject-specific composition of soft-tissue compartments to elucidate their contribution to vibrations.

## Introduction

When a runner contacts the ground, the impact forces during landing initiate vibrations of the soft-tissue compartments of the lower extremities [1, 2]. As the muscle’s vibrations cannot be measured separately from the surrounding tissue, the term soft-tissue includes the muscle itself, subcutaneous fat, ligaments, tendons, and the skin that make up the measured compartment. Its vibrations can be affected by (a) changing mechanical properties of the individual soft-tissue compartments [1] and/or (b) changing the characteristics of the input signal [3].

The mechanical properties of soft-tissue compartments depend on two possible factors, the individual anthropometrics and the muscle activations. The anthropometrics are a given for each subject. They are speculated to influence the soft-tissue vibration propagation tremendously [3] and were shown to affect the preferred adaptation strategy (damping or shifting the frequency) [4]. However, to actively change vibrations during running in preparation for the impact, an anticipatory change in muscle activation is necessary [5], called pre-tuning. These changes in muscle activation will alter the metabolic cost while running and would energetically not be advantageous [6, 7]. Previous studies showed that pre-tuning could alter the vibration frequency and the damping coefficient of soft-tissue [5, 8–10]. It was also shown that tensing muscles reduces skin-movement artefacts [11] as they are major contributors to skin motion [12].

In running, the input signal can be influenced by multiple factors [3, 13], such as footwear and landing geometry and running speed. Adjusting footwear characteristics represents a simple option to change the input signal [14–17] and alter soft-tissue vibration characteristics for a given individual. Previous studies [8, 10, 18, 19] have investigated how footwear affects soft-tissue vibrations and muscle pre-tuning by altering footwear characteristics. Footwear has shown to changes input signal characteristics in some cases [10, 18, 20] but not in others [19]. Overall, footwear that reduces soft-tissue vibrations in their amplitude (damps vibration) or shifts the vibration frequency away from the natural soft-tissue frequency were suggested to be beneficial for the athlete [3]. Based on a recently performed study [17], changes in midsole viscoelasticity might affect the damping of soft-tissue vibrations more than shifting their vibration frequency. A modelling study confirmed that adjusting shoe hardness and pre-tune the muscles can correctly simulate soft-tissue vibrations during running [21].

Although previous studies have contributed significantly to understanding how footwear affects soft tissue vibrations and muscle pre-tuning, some concerns remain. The samples of most studies were typically small (n between 8 and 20) and homogeneous (male, lean runners). This limitation might affect the possibility to translate previous findings to the general running population. Soft tissue properties can vary tremendously across age, sex and body type of the participants. Nevertheless, including a broader sample of participants to quantify muscle activation and soft-tissue acceleration is challenging due to the known risk of subcutaneous soft-tissue layers impacting the quality of muscle activation measurements [22]. This challenge needs to be considered prior o determining the sample size for the present study. Due to a high estimated exclusion of muscle activation raw data, the total number of collected subjects must be increased. Furthermore, it is unknown how subcutaneous soft-tissue layers affect soft-tissue acceleration data. It seems likely, that more subcutaneous soft-tissue masks the actual muscle acceleration by either enhancing the vibrations or counteracting them. Since muscle acceleration cannot be separately investigated from subcutaneous soft-tissue acceleration *in vivo*, vibration patterns that seem unlikely to occur in healthy participants need to be excluded from the data set. Despite these logistic challenges, it is essential to quantify the effects of footwear on muscle activation and soft-tissue accelerations across runners with big differences in their sex, age and anthropometrics. This approach will allow for a detailed understanding of the mitigation of soft-tissue vibrations during running in the general population.

Therefore, the purposes of this study were (a) to determine the vibration and pre-tuning responses of the gastrocnemius medialis muscle across a wide population of participants running in a soft and hard shoe, (b) to form groups according to the damping coefficient and (c) to investigate the anthropometrical differences between these groups.

## Methods

The ethics committee of the University of Calgary (REB18-0235) approved the protocol of this study and written informed consent was obtained of all participants. In total, 100 (42 females) active individuals who were free from injury participated in this study. They were screened concerning the quality of their muscle activation and acceleration data in two shoe conditions (similar to the geometry of the Salomon Sonic RA 2018, Salomon SAS, Annecy, France). Thirty-two subjects (15 females) showed clean muscle activation (S1 Fig) and acceleration data (outside the physiological range of 10 s^-1^ ≤ c ≤ 110 s^-1^). They were used for further analysis.

### Properties of the shoes

**Fig 1:**
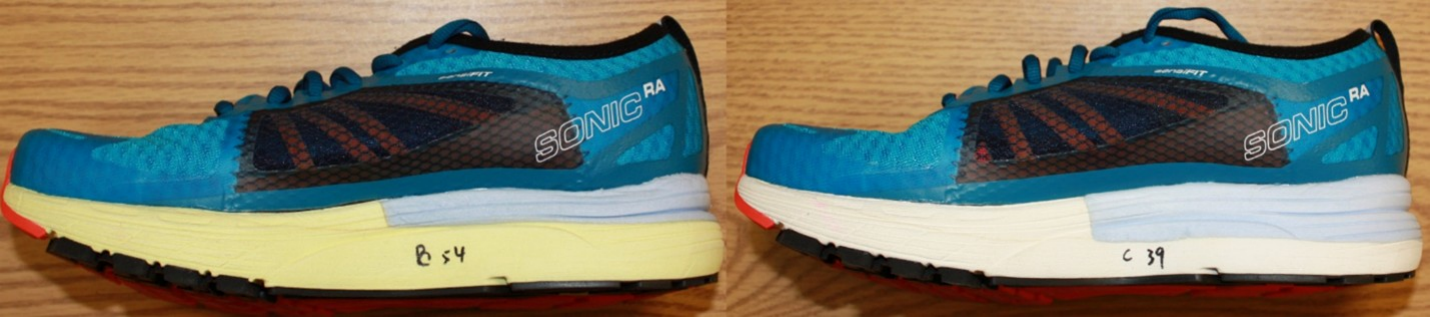
Testing shoes. The hard shoe can be seen on the left side, and the soft shoe is on the right side. The overall heel thickness remains the same across shoes, while only the hardness of the EVA foam (yellow/white material) and the thickness of the heel layer (blue material) differed.

The two tested shoe conditions differed only in their midsole hardness. Both shoes were identical in their overall heel thickness (Fig 1). The hard shoe consisted of a hard ethylene-vinyl acetate (EVA) foam (64 shore C) and a thin heel layer (5 mm), while the soft shoe consisted of the soft EVA foam (56 shore C) and a thick heel layer (18 mm). This resulted in one very hard shoe (shoe H) and one very soft shoe (shoe S).

### Testing procedure

Participants ran on a treadmill (Quinton Q65, Quinton Instruments Co., Seattle, WA). For the 5-minute warm-up, each participant ran in their own shoes at a self-selected comfortable speed during which maintaining a conversation was possible. Self-selected speed was chosen to ensure a wide selection of runners regarding their fitness levels and minimize fatiguing effects. At the end of the warm-up, the selected speed was used to test the hard and soft shoe for the recorded running trials. Only natural heel strikers were included in the study. Participants performed two 5-minute running trials in the two shoes in a randomized order.

During the running trials, electromyography (EMG) and 3D acceleration were measured from the right gastrocnemius medialis (GM) muscle using the Trigno Wireless system (sampling rate: 2000 Hz (EMG) & 148 Hz (accelerometer), Delsys Inc., Natick, USA). The natural vibration frequency of soft-tissue vibrations was reported to be within a range between 7 to 40 Hz for the triceps surae muscle group [23], which would be sufficiently sampled with 148 Hz according to Nyquist theorem. The skin was shaved and cleaned before placing the sensor (mass: 14.7g) according to the SENIAM guidelines with its x-axis along the longitudinal axis of the shank [24]. An additional Trigno sensor was placed on the heel of the right foot to determine the time of heel strike following previous approaches [25, 26]. Sensors were fixed and taped with cover-roll tape (Beiersdorf AG, Hamburg, Germany). All EMG and vibration data were processed and analyzed using Matlab software (Version 2018b, The MathWorks, Natick, USA).

#### EMG analysis

A wavelet analysis technique with 20 non-linearly scaled wavelets (centre frequencies ranging from 2.5 Hz to 247.5 Hz, scaling of 1.3) was used to resolve the EMG into time-frequency space [27]. Wavelets below 10 Hz centre frequencies were neglected due to the high risk of containing movement artefacts [28]. The EMG signals were analyzed for a time window of 100 ms before heel strike to determine the pre-activation of the muscle. All wavelet transformed EMG signals were normalized to the total intensities above 100 Hz [29]. The EMG signals were averaged across the last 100 steps of each 5 min running trial. EMG total intensity was defined as the sum of EMG intensities and was analyzed for three frequency bands: low (12.5–60 Hz), medium (60–120 Hz), and high (120–250 Hz).

#### Soft-tissue vibration analysis

Axial acceleration data was analyzed for a time window of 100 ms after heel strike to capture the entire propagation soft-tissue vibrations after touchdown [23, 30]. The signals were filtered with a wavelet high-pass filter of 7 Hz (mode 30) to remove movement artefacts. A fast Fourier transformation was performed on the zero-padded acceleration data to obtain a power spectrum with a 1 Hz resolution. The dominant vibration frequency for each step (maximum value of the power spectrum) for the GM was determined within the range from 7 Hz to 40 Hz [23]. Dominant frequencies were then averaged across the last 100 steps of each condition.

**Fig 2:**
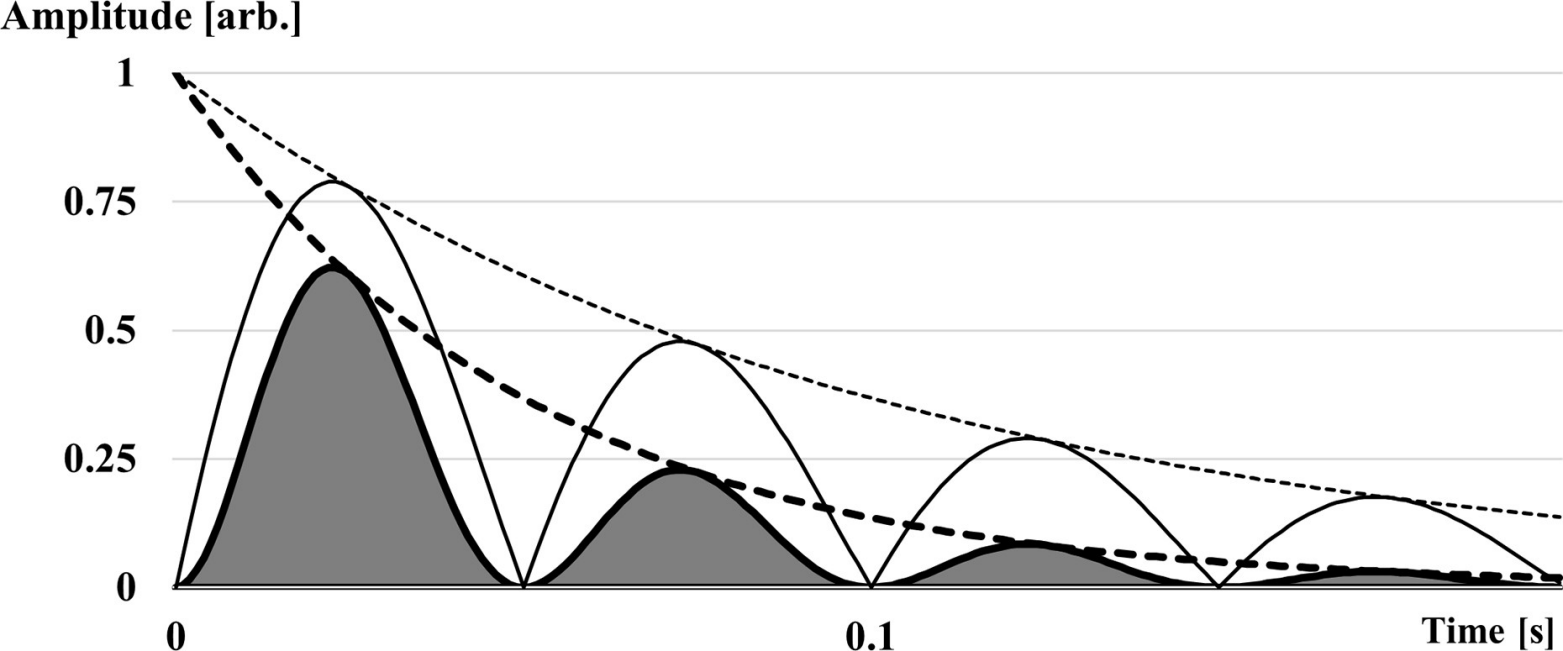
Illustration of damped oscillations. Schematic representation of the model with a dominant vibration frequency of 10 Hz and a damping coefficient c = 20s^-1^. The damped absolute signal (thin solid line) with its exponential decay (thin dashed line) and the signal power (squared signal; thick solid line) with its exponential decay (thick dashed line) is illustrated. The grey area indicates the absorbed energy of the signal (sum of squared absolute amplitude or power-time integral).

In this study, signal power was determined as the squared acceleration signal over 100 ms after heel strike [23, 30]. Dividing signal power by the length of the signal was considered redundant as the same window size was chosen across all trials and participants. The energy absorbed by the body was defined as being proportional to the time integral of the power-time curve (sum of squared absolute amplitudes). To account for differences in amplitudes due to potential running speed effects [18, 19], the power-time curve was normalized to its maximum before energy calculations were performed. By definition, damping represented the decay of the initial power over time and was expected to follow an exponential decay, exp(-c * t), where c described the damping coefficient and t the time (Fig 1; [1, 25, 28]). The decay of the actual vibration signal decayed with exp(-c/2 * t) (Fig 2). A small damping coefficient indicated less damping. Since signal power did not show a perfectly exponential decay over time, a model computation was developed to approximate the damping coefficient. The dominant vibration frequencies (7–40 Hz) were used to create modelled, damped (damping coefficient between 5 to 120 s^-1^) sinusoidal vibration signals (amplitude = 1). The power-time curve was created for every modelled signal from which the absorbed energy was derived, as mentioned above. The experimental vibration data’s absorbed energy, and dominant vibrations frequency was then matched with the most similar absorbed energy and dominant vibration frequency values from the modelled data. This procedure resulted in a damping coefficient assigned for each step and shoe condition for a given dominant vibration frequency and absorbed energy value. The matching process was repeated for the last 100 steps of each shoe condition before being averaged. Despite the model not being specifically validated, its calculation obtained comparable values to other studies [19, 23] even when accelerometers did not sample at high rates (< 1000 Hz).

#### Anthropometrics

Height, weight, Body Mass Index (BMI), and skinfold measurements [31] were performed. From skinfold measurements regarding the right ankle girth, distal and proximal calf-length, right knee girth and proximal and distal calf skinfold, total calf volume, total fat-free calf volume, and estimated calf mass were calculated using pre-defined equations of anthropometry [31, 32] (S2 Fig and S1 File).

### Statistical analysis

Shapiro Wilk tests were performed for the following variables: damping coefficient, dominant vibration frequency, total intensity of low, medium and high frequency bands and anthropometrical variables. None of the variables were normally distributed (p ≤ 0.01, α = 0.05). Therefore, only nonparametric tests were performed to test for differences.

Wilcoxon (W) tests were run to determine significant differences in damping coefficients, change of muscle activation and vibration frequencies between shoe conditions. Groups of participants were formed based on the shoe condition that yielded a significantly lower average damping coefficient in within-subject comparisons across 100 steps using the Mann-Whitney-U (MWU) test. Kruskal Wallis (KW) tests were performed to determine statistical differences between the three groups regarding running speed and anthropometrics. The level of statistical significance for each test was set to α = 0.05.

## Results

Participants were on average 27 years (range: 18 to 49 years) with a height of 173.4 cm (149.5 to 194.0 cm), body mass of 71.7 kg (53.0 to 109.0 kg) and a BMI of 23.8 (19.3 to 33.6). The average treadmill speed was 8.4 km/h (6.1 to 11.7 km/h).

### Vibration and pre-tuning response

Dominant vibration frequencies (p_W_ = 0.804), damping coefficients (p_W_ = 0.722) or pre-heel strike muscle activities in the GM for all three frequency bands (low p_W_ = 0.125, medium p_W_ = 0.734 and high p_W_ = 0.276) were not significantly different between the soft and hard shoe (Table 1).

**Table 1:** Vibration characteristics.

Variable	Hard Shoe	Soft Shoe
**f [Hz]**	10.5 ± 2.7	10.4 ± 3.0
**c [s** ^**-1**^ **]**	60.7 ± 21.7	57.5 ± 23.1
**Low [mV]**	100.8 ± 34.3	99.1 ± 36.2
**Medium [mV]**	126.4 ± 22.0	125.9 ± 25.3
**High [mV]**	86.5 ± 15.5	85.6 ± 17.2

Mean ± std for dominant vibration frequencies (f), damping coefficients (c) and muscle activity in three frequency bands (low, medium and high) when running in a soft and hard shoe.

**Fig 3:**
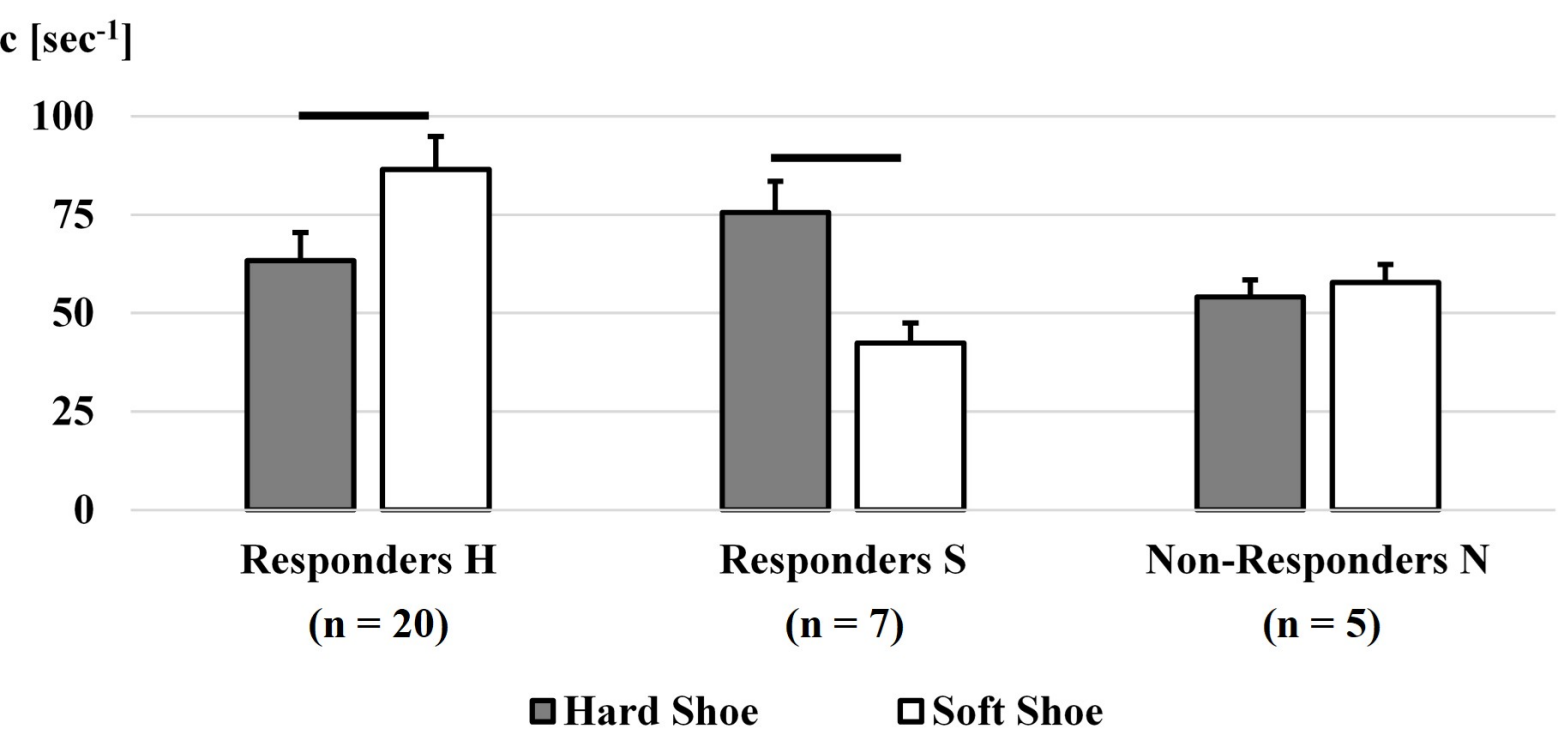
Group comparison of damping coefficient. Mean ± se of damping coefficients (c) per group according to shoe conditions (hard and soft) with the minimal damping coefficient for the gastrocnemius medialis. Significance is indicated as a black bar.

On a subject-specific level, the changes in damping coefficients (-43 ≤ Δc ≤ 65 s^-1^) were larger than the changes in dominant vibration frequencies (-3 ≤ Δf ≤ 4 Hz) when switching from a hard to a soft shoe (Fig 3).

### Grouping according to damping coefficient

**Fig 4:**
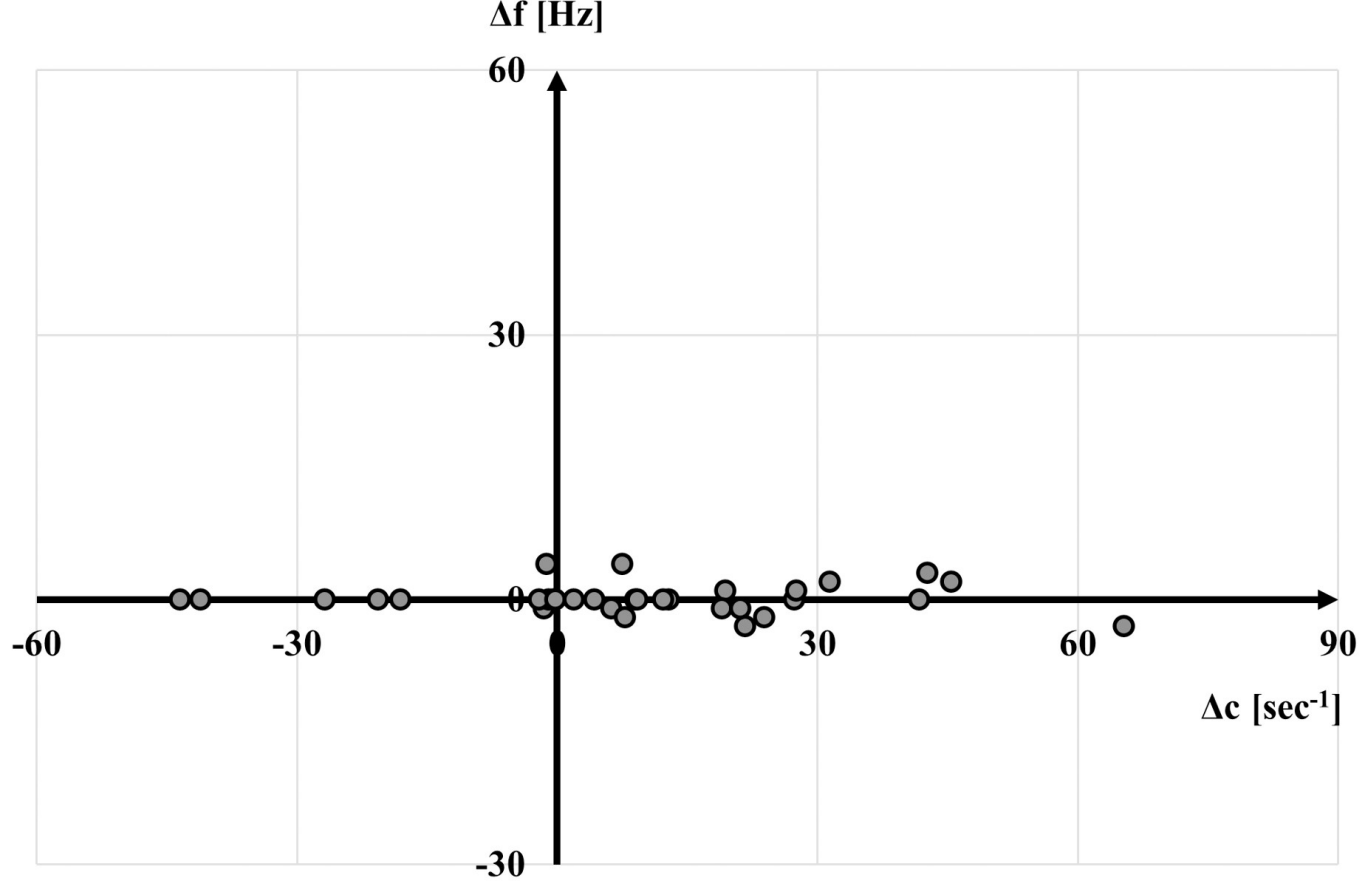
Differences (shoe hard–shoe soft) between two shoe conditions regarding dominant vibration frequency (f) and damping coefficient (c) in the gastrocnemius medialis (n = 32).

Three groups were formed according to the damping coefficient responses in each shoe condition (Fig 4). The biggest group of participants (20/32 = 62.5%), responders H, contained participants who had a significantly lower damping coefficient in the hard shoe (p_MWU_ ≤ alpha). The second biggest group (7/32 = 21.8%), responders S, showed a significantly lower damping coefficient in the soft shoe (p_MWU_ ≤ alpha). The third group (5/32 = 15.6%), non-responders N, reported no significant changes (p_MWU_ > alpha) in their damping coefficients across shoe conditions.

### Differences between groups

Pre-heel strike muscle activities in the GM (low p_KW_ ≥ 0.787; medium p_KW_ ≥ 0.981; high p_KW_ ≥ 0.699) and dominant vibration frequencies (p_KW_ ≥ 0.486) did not show differences between the three groups (Table 2). Anthropometric measurements (p_KW_ ≥ 0.483) and running speeds (p_KW_ ≥ 0.217) were not significantly different between the three groups (Table 2).

**Table 2:** Vibration characteristics and anthropometrics after grouping according to damping coefficient.

Variable	Responders H (n = 20)	Responders S (n = 7)	Non-responders N (n = 5)	p-value
**f**_**H**_ **[Hz]**	10.4 ± 2.4	12.4 ± 3.7	8.8 ± 0.8	0.486
**f**_**S**_ **[Hz]**	10.4 ± 2.6	11.3 ± 4.5	9.2 ± 1.3	0.729
**Low**_**H**_ **[mV]**	108.8 ± 27.7	102.0 ± 20.8	122.0 ± 33.4	0.7866
**Low**_**S**_ **[mV]**	109.0 ± 33.0	98.0 ± 22.2	117.0 ± 31.2	0.738
**Medium**_**H**_ **[mV]**	131.5 ± 26.2	122.8 ± 17.6	124.2 ± 28.0	0.927
**Medium**_**S**_ **[mV]**	129.0 ± 27.9	123.5 ± 18.2	127.6 ± 33.1	0.981
**High**_**H**_ **[mV]**	87.0 ± 13.2	84.8 ± 13.0	79.7 ± 15.9	0.874
**High**_**S**_ **[mV]**	85.4 ± 15.3	85.1 ± 14.2	78.1 ± 15.3	0.699
**Speed [km/h]**	7.4 ± 1.6	8.7 ± 0.9	8.5 ± 1.4	0.217
**Height [cm]**	172.7 ± 10.5	176.2 ± 7.9	171.5 ± 6.3	0.696
**Body Mass [kg]**	73.3 ± 17.3	71.3 ± 14.4	72.3 ± 12.0	0.831
**BMI**	24.7 ± 4.6	23.2 ± 3.4	23.9 ± 4.3	0.807
**Total V [cm** ^**3**^ **]**	4161.0 ± 793.3	3894.3 ± 695.5	4159.6 ± 998.0	0.716
**Ffree V [cm** ^**3**^ **]**	3611.0 ± 541.7	3434.8 ± 863.1	3679.1 ± 908.3	0.483
**Calf Mass [kg]**	5.3 ± 0.9	5.0 ± 1.0	5.3 ± 1.3	0.691

Mean ± std for dominant frequencies (f), muscle activation for all three frequency bands (low, medium and high), running speed and anthropometrics for each shoe condition (soft, S, and hard H) across all three groups. Calculations of skinfold measurements included total calf volume (Total V), fat free calf volume (Ffree V) and soft-tissue mass of the calf (Calf Mass). P-values were obtained for comparisons of the three groups.

## Discussion

The objectives of this study were (a) to determine the vibration and pre-tuning responses of the GM muscle across a broad population of participants running in a soft and hard shoe, (b) to form groups according to the damping coefficient and (c) to investigate the anthropometrical differences between these groups.

### Vibration and pre-tuning response

Across all participants, the vibration and pre-tuning responses for the GM remained similar when switching from a soft to a hard shoe. The average dominant vibration frequency across all participants remained low (~10 Hz). The average damping coefficient across all subjects was strong, and only a few oscillations resulted from the impact after touchdown [23]. On a subject-specific level, it was shown that the damping coefficient changed much more drastically across footwear conditions than the dominant vibration frequency. This finding is in agreement with previous studies [19, 20]. The significant changes in the damping coefficient indicate that the damping coefficient is more sensitive to altered footwear characteristics than the dominant vibration frequency. Surprisingly, the change in damping coefficient was not accompanied by a change in muscle pre-tuning, which was reported by other studies [4, 8]. One possible explanation for this might be the subtle change in footwear which did not require adjustments in muscle activation to prepare for the impact. Furthermore, dominant vibration frequencies were on the lower spectrum of previously reported natural soft-tissue frequencies. They might not have been in the area to cause damage to the tissue in the first place. In summary, the musculoskeletal system primarily adjusted the damping coefficient when changing footwear.

### Grouping according to damping coefficient

Since changing the damping coefficient was the primary response to footwear alterations, participants were grouped according to the shoe condition with the lowest damping coefficient. A low damping coefficient was considered to be beneficial from a vibration attenuation aspect. It means that only little damping is required to minimize the vibrations in their amplitude and duration. On the contrary, a high damping coefficient implies that a lot of damping is needed to attenuate soft-tissue vibrations. Most participants (62.5%) showed significantly lower damping coefficients in the hard shoe (responders H) compared to the soft shoe (responders S). Therefore, shoes resulting in no or little damping might be considered beneficial as only minor adjustments were required to minimize potentially harmful soft-tissue vibrations [33]. Less soft-tissue vibrations were also suggested to be more comfortable [3, 33], which would be advantageous for footwear companies when designing novel running shoes. In summary, the majority of participants benefitted from a hard midsole while running.

### Differences between groups

Differences in damping coefficients could be due to multiple factors such as changing muscle activity [8] and anthropometric characteristics affecting the damping behaviour [34].

Damping soft-tissue vibrations actively were assumed to be achieved by increased muscle pre-tuning [8, 9]. The overall pre-heel strike muscle intensity of low, medium and high frequency bands remained similar across footwear conditions and groups. This might be because of the small differences in midsole hardness between the tested shoe conditions. Therefore, there was no different pre-tuning strategy evident between both shoes, which is supported by previous findings [19]. Consequently, one must look for other explanations of how the exact mechanism of energy dissipation of the vibration occurs *in vivo*. Little is known how soft-tissues such as subcutaneous tissue, tendons, ligaments and muscles dissipate energy in the human body during locomotion. This knowledge would be crucial to understand how different damping strategies can develop without changes in pre-muscle activation. However, similar muscle activation could be interpreted as similar metabolic costs while running [7]. Therefore, footwear resulting in less soft-tissue damping and similar metabolic cost might be considered advantageous from a performance, comfort and injury perspective.

Anthropometrics and fitness levels were speculated to influence the damping behaviour of soft-tissue vibrations [34, 35]. A spring-like oscillator’s frequency is inverse proportional to the square root of the mass. Therefore, heavier individuals would be expected to show lower dominant vibration frequencies. Fit people with less body mass and very likely with a lower BMI would be expected to display a higher dominant vibration frequency. Despite no differences in dominant vibration frequency, responders H (lower damping coefficient in hard shoe) showed a trend of a slightly greater BMI (shorter and heavier), slower running speed and greater calf volume and soft-tissue mass compared to responders S (lower damping coefficient in a soft shoe). This is especially important, as the additional mass is not distributed homogenously around the limb and might change the vibration and damping characteristics tremendously. Future studies should further focus on the interaction between composition and distribution of soft-tissues and damping variables.

In summary, muscle-tuning changes were not detected by muscle intensity measurements when switching from a soft to a hard running shoe. Instead, anthropometrical characteristics might passively damp soft-tissue vibrations. Heavier and less trained individuals might benefit from a hard shoe when running on a treadmill.

### Limitations

This study assumed that the soft-tissue compartments behave like simple mass-spring models. Additionally, it was assumed that there is one single characteristic for a given soft-tissue compartment. We only analyzed the axial direction, but research showed that vibration characteristics could also play a substantial role in other directions [5]. Both assumptions are simplifications. Soft-tissues have different compartments with potentially different vibration frequencies and damping characteristics [36].

Kinematics were not quantified in this study. Footwear could have affected running kinematics, in particular landing geometry. Different landing geometries would need to be introduced by other footwear characteristics and/or muscle pre-activation. Since there were no significant differences in muscle activation between footwear conditions and the differences between the footwear conditions were subtle as they had identical heel geometry, differences in running kinematics seemed unlikely.

The input signal was not directly measured in this study, as the data collection took place on a non-instrumented treadmill. For a subset of ten participants, additional force data were collected using a force platform (2400 Hz sampling rate, Kistler Instrument AG, Winterthur, CH), which was embedded within the laboratory floor. Timing lights controlled for the participant’s running speed for over ground running trials. The required speed was considered constant within a 5% margin of the participant’s self-selected speed on the treadmill. Participants completed three overground running trials in each shoe condition. The input frequency was computed on the passive peak of the ground reaction force [13]. One participant was excluded due to a loose sensor. Across the remaining nine participants, the input frequencies between the two shoe conditions were not significantly different (mean ± std: hard shoe 15.8 ± 1.5 Hz, soft shoe15.9 ± 2.1; p_Wilcoxon_ = 0.16) and stayed on average within a range of 2.6 Hz within a participant. Despite treadmill running being different from overground running, the comparison of input signals across footwear holds true as a paired-comparison design, independent of the running surface. In summary, the input frequency does not account for the differences in damping coefficients that were shown for participants when changing footwear.

Damping was calculated based on a sinusoidal model calculation and/or the exponential decay of power over time [1, 5, 23]. These procedures required the peaks of the signal power curve to be sorted in a decreasing order, which was not evident in the present study. This might be due to the large range of running speeds and body types of the participants, which yielded a wide range of soft-tissue vibration patterns. Potential running speed effects were accounted for by normalizing the power-time curves as described in the method section. The modelled damping coefficient values were in accordance with previous studies [19, 23]. Future studies should expand the current model to multiple sinusoidal signals similar to previous work [37], for which a higher sampling rate of acceleration signals would be required.

Increased subcutaneous soft-tissue layers and excessive sweat impact the quality of muscle activation measurements such as surface EMG [22] and potentially acceleration signals. Therefore, obtaining clean muscle activation and acceleration data is extremely challenging in biomechanical set-ups when a wide range of body types and fitness levels is tested. The current study tested a large population of participants (n = 100) and pre-screened their EMG and acceleration signals to ensure clean data is available for further analyses (n = 32).

## Conclusion

Soft-tissue vibrations in participants with a wide range of body types are mainly affected by their damping coefficients when footwear changes during treadmill running. Damping coefficients were successful in determining three groups according to damping responses in the tested footwear conditions. A lower damping coefficient at the same frequency suggests that less energy dissipation is necessary for the corresponding shoe to protect soft-tissues from vibrating. Most participants might benefit from a hard shoe (lower damping coefficient, similar pre-tuning). Pre-tuning of the muscle does not seem to be the only mechanism that adjusts the energy dissipation and thus the damping coefficient. It remains unknown how exactly vibration energy is dissipated *in vivo* during treadmill running. The results for anthropometrics and their effect on vibration damping were insignificant but showed a trend. It is speculated that calf segment volume and mass might affect soft-tissue vibration responses in the triceps surae. Future studies should further investigate the effect of soft-tissue composition on vibration responses.

## Supporting information

S1 Fig Demonstration of exclusion criteria for EMG signals. The left column (A & C) represents an example of one good subject/condition, whereas the right column (B & D) represents an excluded subject/condition. First, intensity wavelet plots (A & B) for the hard and soft shoes were visually inspected by two independent investigators for artefacts such as high activity prior to heel strike (HS). If two investigators determined artefacts, the participant was excluded. Second, participants were excluded if the overall intensity (sum of all intensities of wavelet plot mean ± std) between the 1^st^ and repeated condition were significantly different (C-D; indicated with black bar, unpaired t-test over 100 steps per condition with alpha 0.05).
(TIF)

S2 Fig Illustration of the anthropometrics. Girth (black solid line), length (solid double arrows) and skinfold (dashed circular with X) measurements that were used to calculate the anthropometrics. The position of the EMG and acceleration sensors (grey rectangles) are also indicated.
(TIF)

S1 File Equations for skinfold measurements. The estimation of segmental mass, volume and fat-free volume were calculated by assuming truncated cones for the calf [31]. The mass of the calf is a function of volume and density [32]. Length (L#), girth (G#), and skinfold (S#) measurements can be seen in S2 Fig.
(PDF)

S2 File Data. This archive contains all the underlying data presented in this publication to follow the methodological steps of this work. DOI 10.17605/OSF.IO/FKBRN, https://osf.io/fkbrn/.
(PDF)
