# Supplementary figures and images for: Soft-tissue vibration and damping response to footwear changes across a wide range of anthropometrics in running
Source: PLoS One. 2021 Aug 17;16(8):e0256296. doi: 10.1371/journal.pone.0256296 (PMC8370632; doi:10.1371/journal.pone.0256296)

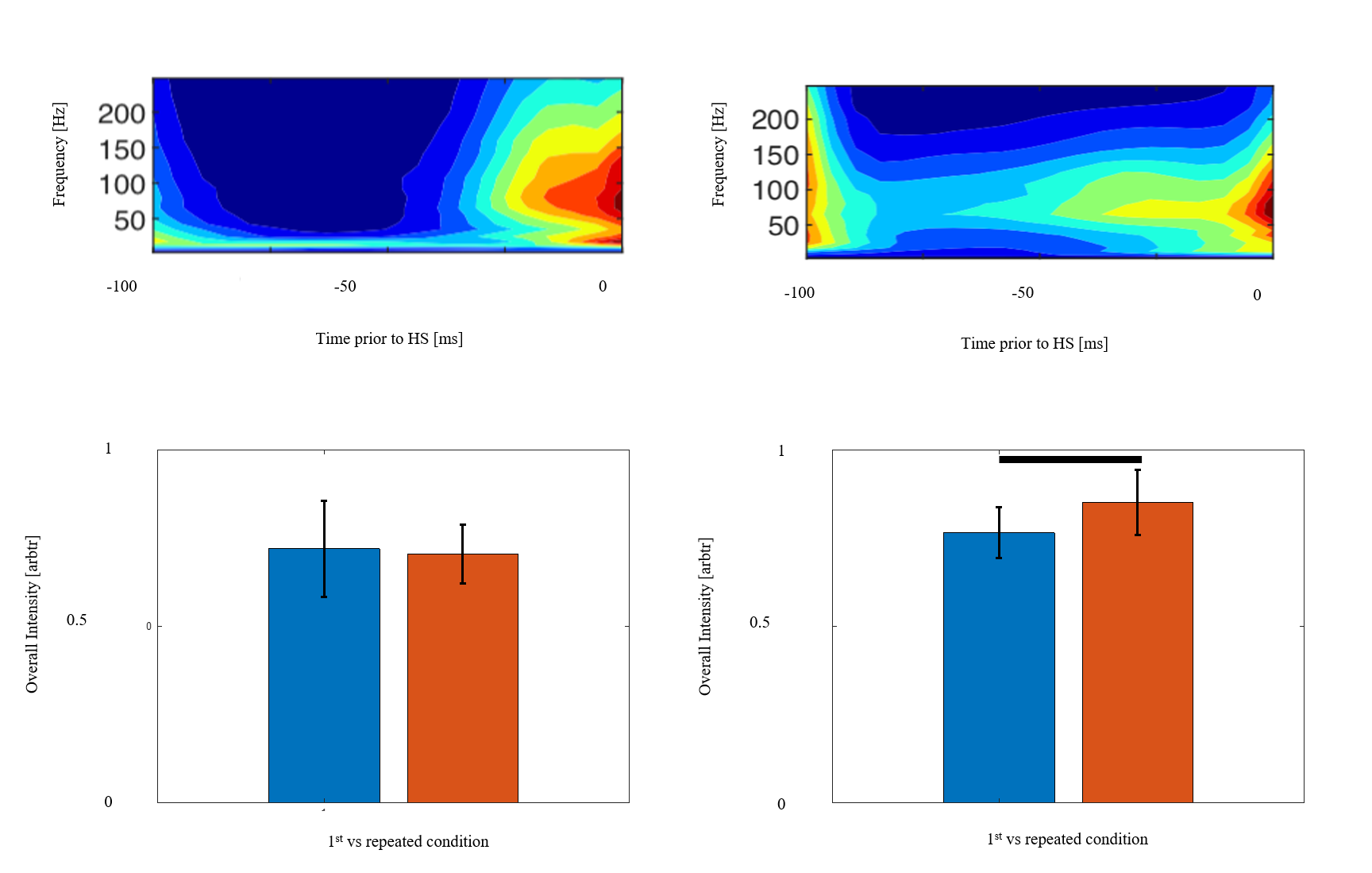

Supplement: S1 Fig — The left column (A & C) represents an example of one good subject/condition, whereas the right column (B & D) represents an excluded subject/condition. First, intensity wavelet plots (A & B) for the hard and soft shoes were visually inspected by two independent investigators for artefacts such as high activity prior to heel strike (HS). If two investigators determined artefacts, the participant was excluded. Second, participants were excluded if the overall intensity (sum of all intensities of wavelet plot mean ± std) between the 1st and repeated condition were significantly different (C-D; indicated with black bar, unpaired t-test over 100 steps per condition with alpha 0.05). (TIF) [file pone.0256296.s001.tif]

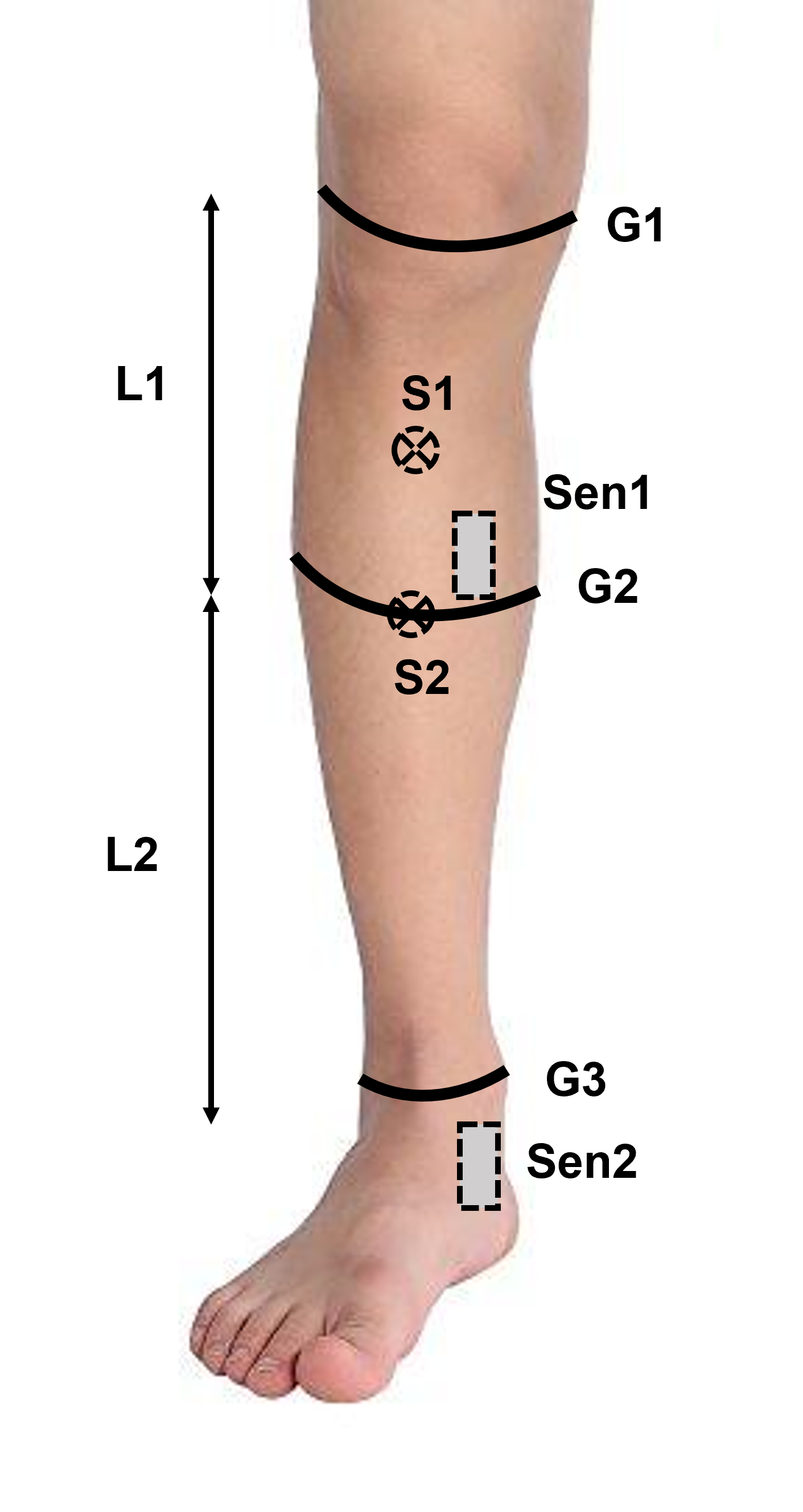

Supplement: S2 Fig — Girth (black solid line), length (solid double arrows) and skinfold (dashed circular with X) measurements that were used to calculate the anthropometrics. The position of the EMG and acceleration sensors (grey rectangles) are also indicated. (TIF) [file pone.0256296.s002.tif]
