# Supplementary material for: Soft-tissue vibration and damping response to footwear changes across a wide range of anthropometrics in running
Source: PLoS One. 2021 Aug 17;16(8):e0256296. doi: 10.1371/journal.pone.0256296 (PMC8370632; doi:10.1371/journal.pone.0256296)
Supplement: S1 File — The estimation of segmental mass, volume and fat-free volume were calculated by assuming truncated cones for the calf [31]. The mass of the calf is a function of volume and density [32]. Length (L#), girth (G#), and skinfold (S#) measurements can be seen in S2 Fig. (PDF) [file pone.0256296.s003.pdf]

Total Calf Volume [V_T; cm^3]

$$V_T = V_P + V_D \quad (1)$$

$$V_P = \frac{L1}{12\pi} * (G1^2 + G2^2 + G1 + G2) \quad (2)$$

$$V_D = \frac{L2}{12\pi} * (G2^2 + G3^2 + G2 + G3) \quad (3)$$

Total fat-free Calf Volume [FFV_T; cm^3]

$$FFV_T = FFV_P + \mathrm{FF}V_D \quad (4)$$

$$FFV_P = \frac{L1}{12\pi} * [(G1 - S1 * \pi)^2 + (G2 - S1 * \pi)^2 + (G1 - S1 * \pi) + (G2 - S1 * \pi)] \quad (5)$$

$$FFV_D = \frac{L2}{12\pi} * [(G2 - S2 * \pi)^2 + (G3 - S2 * \pi)^2 + (G2 - S2 * \pi) + (G3 - S2 * \pi)] \quad (6)$$

Total Calf Mass [M_T; kg]

$$M_T = M_P + M_D \quad (7)$$

$$M_P = V_P + \frac{D_P}{1000} \quad (8)$$

$$M_D = V_D + \frac{D_D}{1000} \quad (9)$$

$$D_P = \frac{4.201}{\frac{\%F_P}{100} + 3.813} \quad (10)$$

$$D_D = \frac{4.201}{\frac{\%F_D}{100} + 3.813} \quad (11)$$

$$\%F_P = \frac{FV_p}{V_P} * 100 \quad (12)$$

$$\%F_D = \frac{FV_D}{V_D} * 100 \quad (13)$$

$$FV_P = V_P + \mathrm{FF}V_P \quad (14)$$

$$FV_D = V_D + \mathrm{FF}V_D \quad (15)$$
