# Supplementary material for: Soft-tissue vibration and damping response to footwear changes across a wide range of anthropometrics in running
Source: PLoS One. 2021 Aug 17;16(8):e0256296. doi: 10.1371/journal.pone.0256296 (PMC8370632; doi:10.1371/journal.pone.0256296)
Supplement: S2 File — This archive contains all the underlying data presented in this publication to follow the methodological steps of this work. DOI 10.17605/OSF.IO/FKBRN, https://osf.io/fkbrn/. (PDF) [file pone.0256296.s004.pdf]

This archive contains all the underlying data presented in this publication to follow the methodological steps of this work. DOI 10.17605/OSF.IO/FKBRN, https://osf.io/fkbrn/
